# Supplementary material for: Tuning magnetoresistance in molybdenum disulphide and graphene using a molecular spin transition
Source: Nat Commun. 2017 Sep 22;8:677. doi: 10.1038/s41467-017-00727-w (PMC5610345; doi:10.1038/s41467-017-00727-w)
Supplement: Supplementary file 1 — Supplementary Information [file 41467_2017_727_MOESM1_ESM.pdf]

Description of Supplementary Files

File Name: Supplementary Information
Description: Supplementary Figures, Supplementary Tables and Supplementary Note

File Name: Peer Review File
Description:

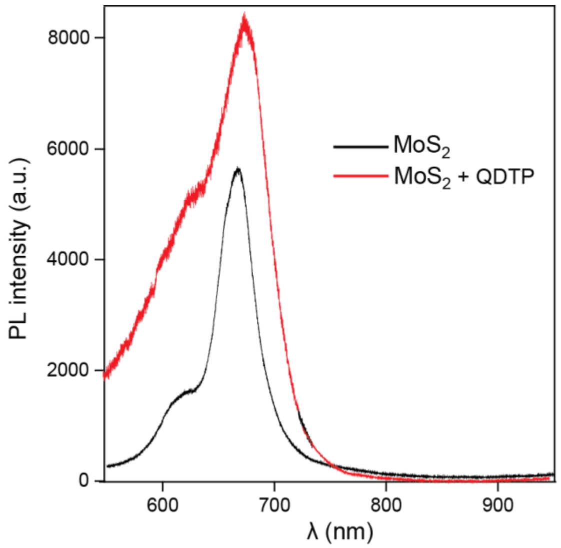

Supplementary Figure 1. **PL spectrum for pristine MoS_2 and hybrid QTDP-MoS_2.** The PL was recorded at 300 K in air with a constant laser excitation power. The PL intensity is much higher for QTDP-$MoS_{2.}$ QTDP molecule does not produce PL in the measured range. The enhanced PL in the case of MoS_2+QDTP is due to the passivation of electron trap sites in MoS_2 by QTDP [1].

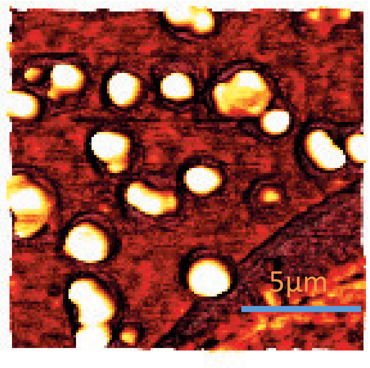

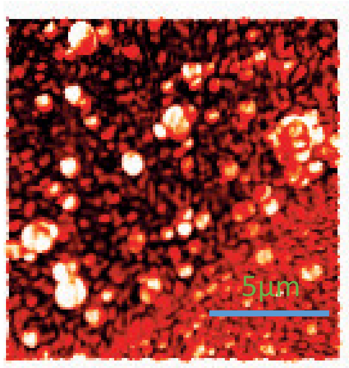

Supplementary Figure 2. **Atomic force microscopy images of the QTDP-2D materials hybrid.** a) AFM image of the graphene - QDTP hybrid. Scale bar is 5 μm. Molecules are adsorbed only on graphene surface. b) AFM image of the MoS_2 - QDTP hybrid. No selective

adsorption of molecules takes place in case of MoS_2.

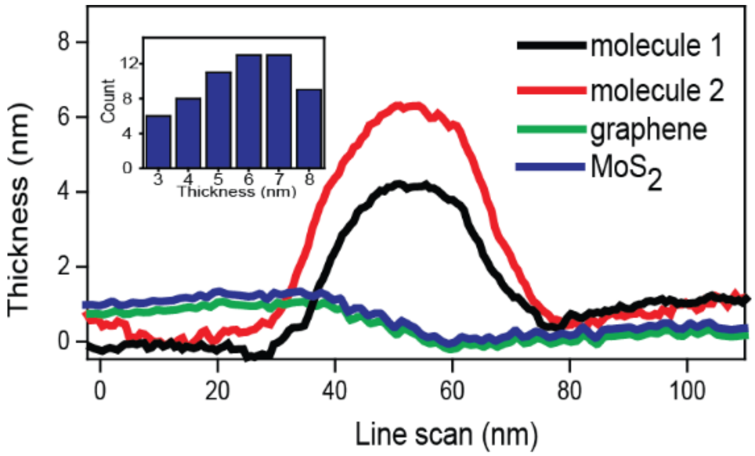

Supplementary Figure 3. **AFM cross-sectional profile**: The thickness of graphene and MoS_2 flakes are 0.85 nm and 0.97 nm, respectively. The size of QDTP molecular assembly could vary from 3 – 6 nm. Inset: Size distribution of molecules over 8 μm^2 area.

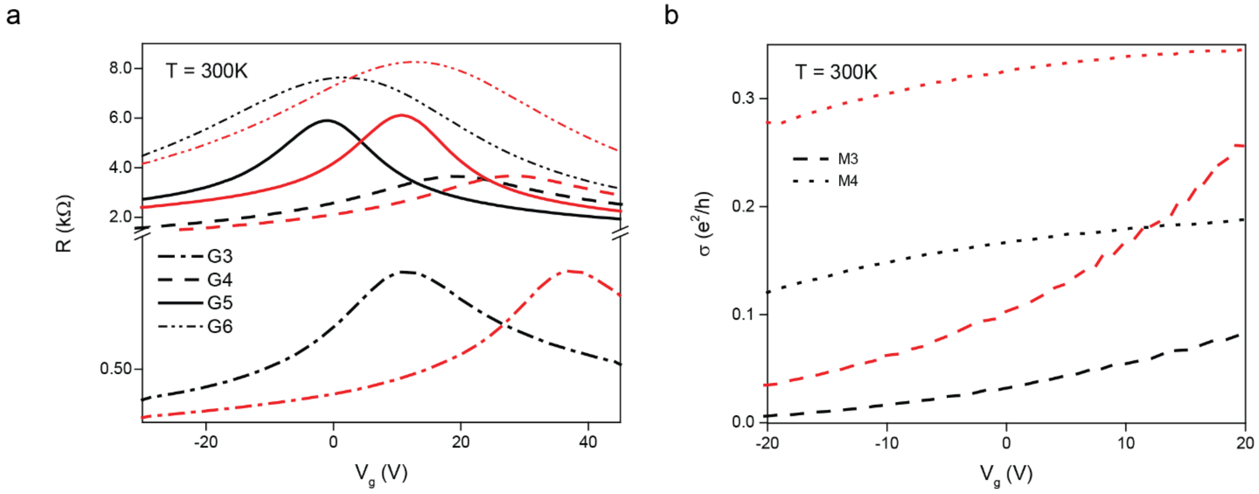

Supplementary Figure 4: **Room temperature transfer curve of different hybrid devices**; **a** pristine graphene (black) and QDTP-graphene (red) devices. A p-type charge transfer has been observed in all samples. **b** Pristine MoS_2 (black) and QDTP-MoS_2 (red) devices. n-type charge transfer has been observed in all samples. While graphene devices are indicated as “G”, MoS_2 devices are termed as “M”. The detailed statistics of these devices are given in the Supplementary Table 1 and 2.

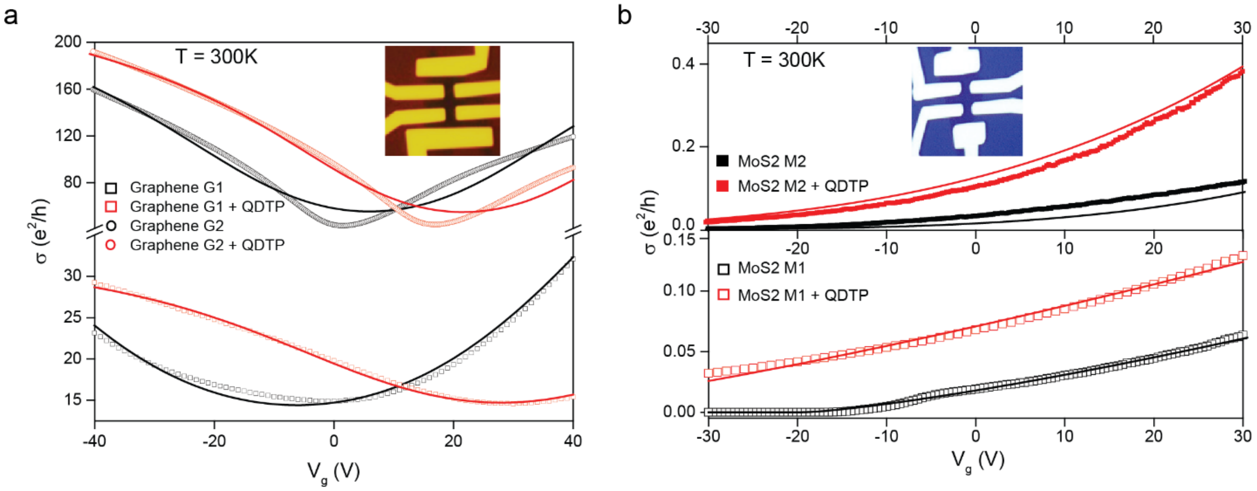

Supplementary Figure 5: **Fitting of transfer curve of two representative hybrid devices**; a Pristine graphene and QDTP-graphene devices. A p-type charge transfer has been observed in both samples. b Pristine MoS_2 and QDTP-MoS_2 devices. n-type charge transfer has been observed.

Sample	Mobility μ (T^{-1} or m^2/V.s)		Doping Δp (10^{10} cm^{-2})
	Pristine	Hybrid	
Sample G1	0.28	0.68	252
Sample G2	2.32	2.51	105
Sample G3	2.91	2.88	162
Sample G4	0.42	0.44	59
Sample G5	0.58	0.71	90

Supplementary Table 1: **Fitting of transfer curves for pritine graphene and graphene-QDTP hybrid devices.** Increment of carrier mobility in graphene after the deposition of QDTP molecule can be explained by compensation of charged impurities. In all samples, p-type doping by QDTP can be noted from the values of charge transfer (Δp).

Sample	Critical exponent<br>β	Doping<br>Δn (10^{10} cm^{-2})
Sample M1	1.29	261
Sample M2	3.80	267
Sample M3	3.10	231
Sample M4	1.03	682

Supplementary Table 2: **Fitting of transfer curves for pritine MoS_2 and MoS_2-QDTP hybrid devices.** n-type doping of MoS_2 by QDTP can be noted from the values of charge transfer (Δn).

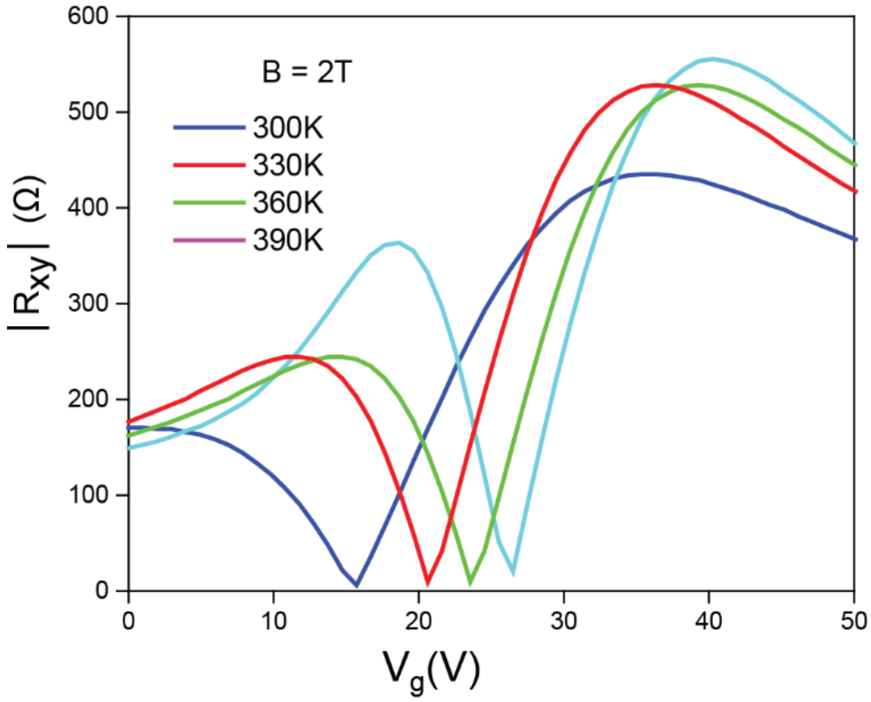

Supplementary Figure 6. **Temperature dependent transverse transfer curve of graphne-QDTP hybrid device.** Gate dependent transverse resistance (R_{xy}) as a function of gate voltages in a perpendicular magnetic field of 2T at selected temperatures (300 K, 330 K, 360 K, 390 K) indicates a gradual shift of charge neutrality point V_D.

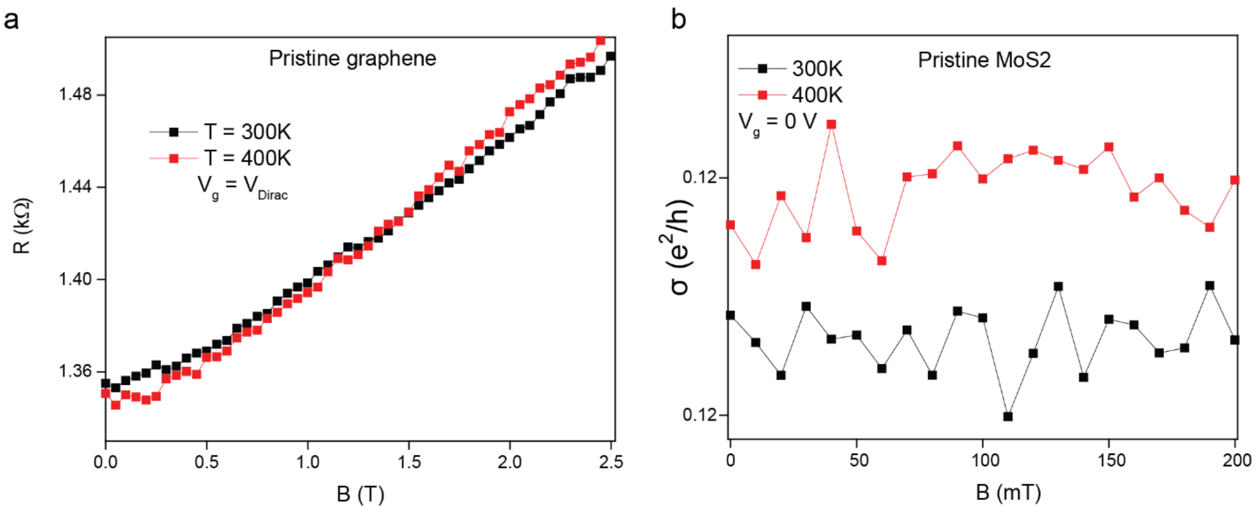

Supplementary Figure 7. **Magentoresistance of pristine 2D devices used in the experiment.** No significant change is observed in case of both devices. While graphene has weak magnetoresistance at room or higher temperature due to presence of impurities (**a**), pristine MoS_2 shows no such change with magnetic field (**b**).

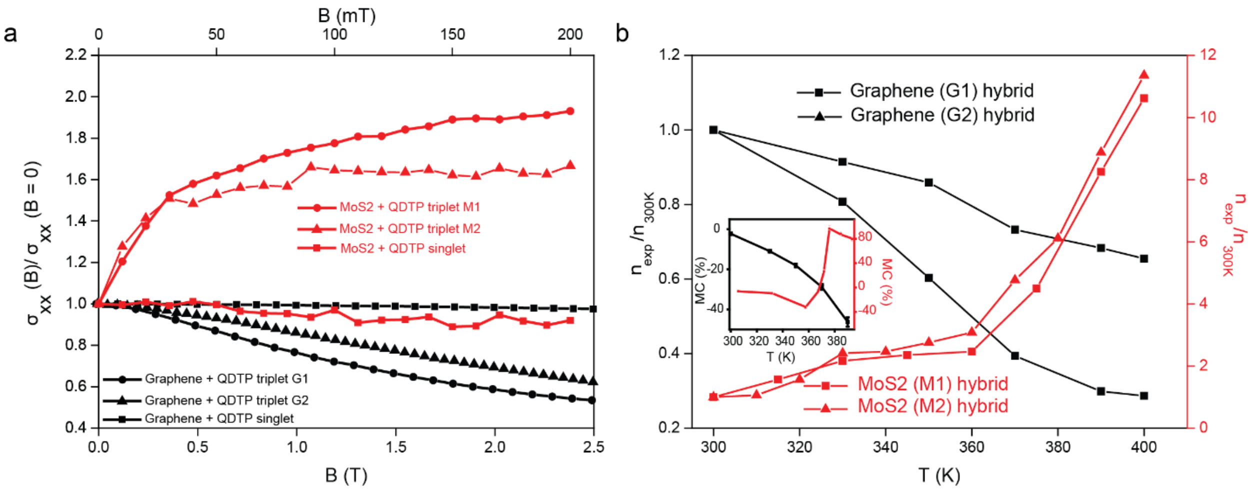

Supplementary Figure 8. **Magnetoconductance and carrier density of two representative samples. a** Graphene-QDTP shows negative MC, while MoS_2-QDTP shows positive MC. Graphene hybrid shows higher magnetoresistance at higher temperature (400 K). **b** MoS_2-QDTP shows a jump in carrier density above spin transition temperature (360 K).

T(K)	$\sigma_{xx,0}(\mu S)$	$\sigma_{xx,1}(\mu S)$	$\mu(cm^{-2}/(V\cdot s))$
300	18 ± 1	205 ± 1	3900 ± 150
330	64 ± 1	145 ± 1	4600 ± 100
350	82 ± 1	124 ± 1	6000 ± 100
370	93 ± 1	97 ± 2	8500 ± 200
390	130 ± 2	59 ± 2	11200 ± 250

Supplementary Table 3. **Fitting parameters of the magneto-conductivity equation σ_{xx} (B) used for graphene-QDTP hybrid.** Above transition temperature, 370K, $\sigma_{xx,0}$ is much larger than $\sigma_{xx,1}$ confirming that magnetoconductivity is better described by $[1+ (\mu B)\ 2]^{-1/2}$ dependence at higher temperatures.

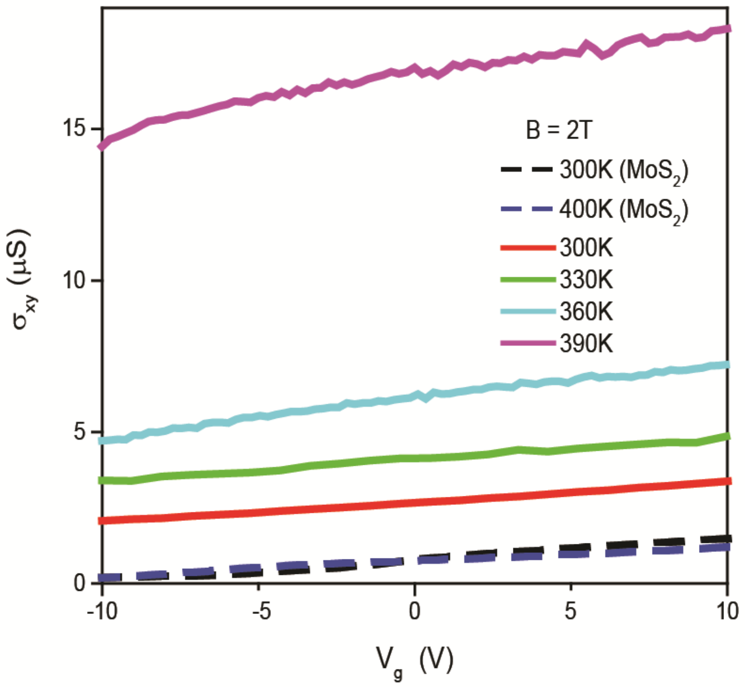

Supplementary Figure 9. **Temperature dependent transverse transfer curve of MoS_2-QDTP hybrid device.** Gate dependent transverse magneto-conductivity (σ_{xy}) as a function of gate voltages in a perpendicular magnetic field of 2 T at selected temperatures (300 K, 330 K, 360 K,

390 K) indicates an increase in overall conductivity after doping. The pristine MoS_2 behavior displayed in dashed lines for reference values.

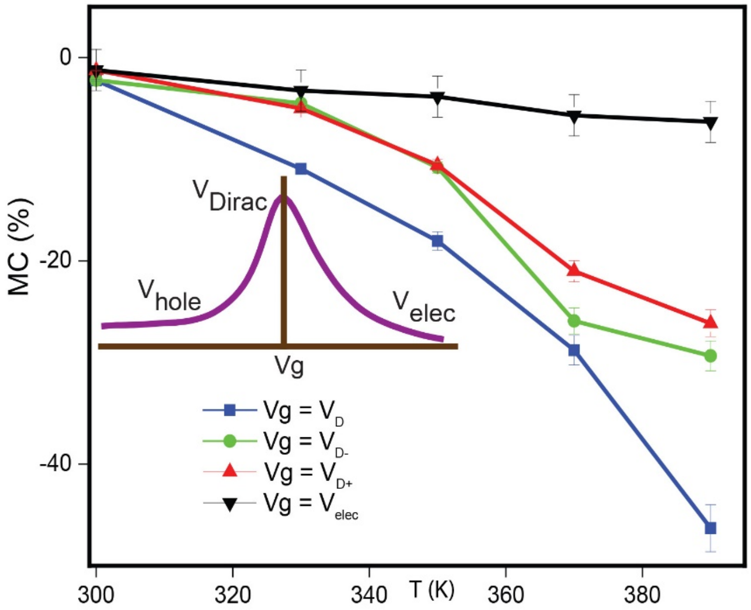

Supplementary Figure 10. **Temperature dependent Magneto-conductance of graphene - QDTP hybrid device.** Magneto-conductance (MC) as a function of temperature for different gate voltages. Highest negative MC at 390K observed at charge neutrality point (V_D). Inset: Intrinsic doping regions of pristine graphene. V_{d+}, V_{d-}, and V_{elec} indicate slightly electron, slightly hole, and electron conduction regions of transfer characteristic curve of hybrid device, respectively.

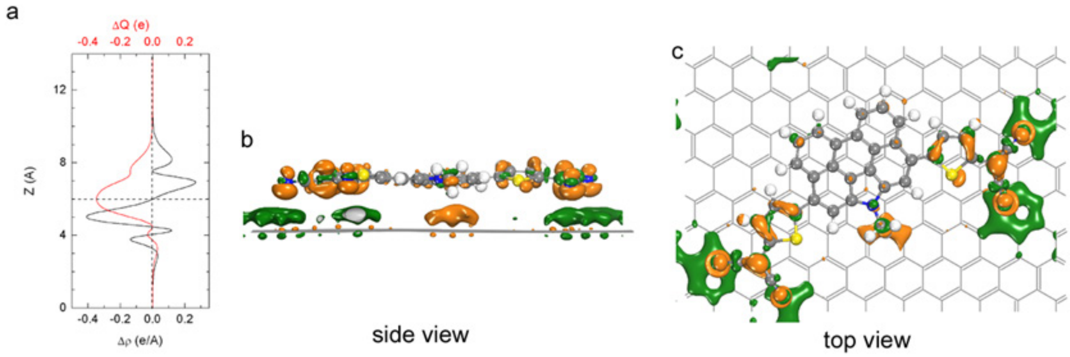

Supplementary Figure 11. **Charge transfer between graphene and QDTP molecule in singlet state in lying-down configuration.** Left panel (a): Plane-averaged differential charge density $\Delta\rho(z)$ (black line), amount of transferred charge ΔQ (red line). The horizontal dashed line in the ΔQ curve defines the maximum amount of transferred charge from the molecule to the graphene. Middle (b) and right (c) panels: Side and top views of the isosurface at value of the 0.004 $Å^{-3}$

(right). The green (orange) color denotes loss (accumulation) of electrons in graphene and QDTP upon combining them. According to ΔQ curve, 0.347e per molecule is transferred from graphene to QDTP.

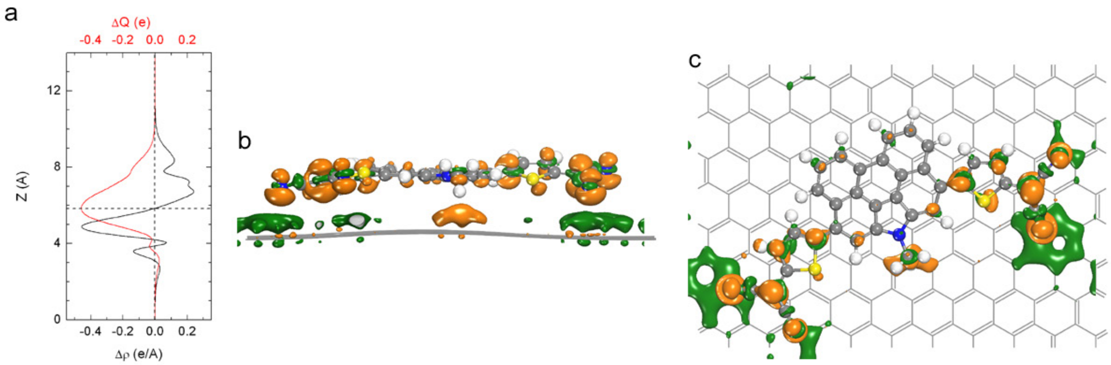

Supplementary Figure 12. **Charge transfer between graphene and QDTP molecule in triplet state in lying-down configuration.** Left panel (a): Plane-averaged differential charge density $\Delta\rho(z)$ (black line), amount of transferred charge ΔQ (red line). The horizontal dashed line in the ΔQ curve defines the maximum amount of transferred charge from the molecule to the graphene. Middle (b) and right (c) panels: Side and top views of the isosurface at value of the 0.004 Å^{-3} (right). The green (orange) color denotes loss (accumulation) of electrons in graphene and QDTP upon combining them. According to ΔQ curve, 0.459e per molecule is transferred from graphene to QDTP.

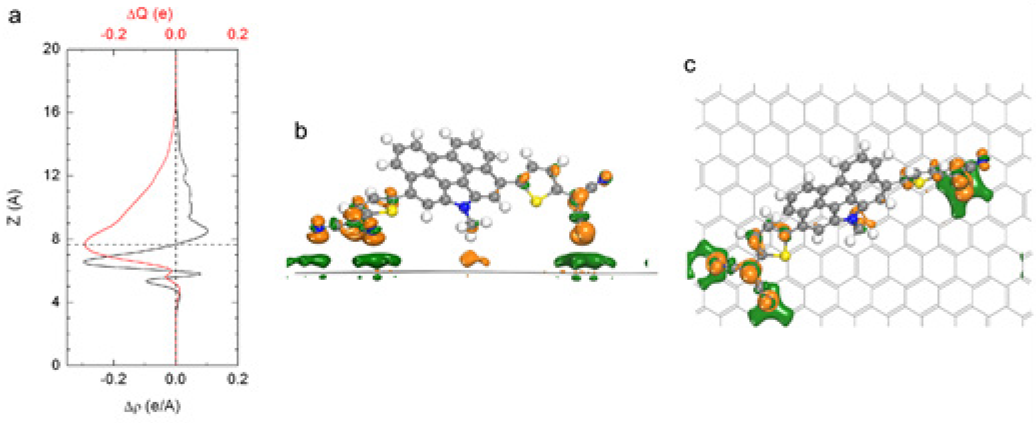

Supplementary Figure 13. **Charge transfer between graphene and QDTP molecule in singlet state in stand-up configuration.** Left panel (a): Plane-averaged differential charge density $\Delta\rho(z)$ (black line), amount of transferred charge ΔQ (red line). The horizontal dashed line in the ΔQ curve defines the maximum amount of transferred charge from the molecule to the graphene. Middle (b) and right (c) panels: Side and top views of the isosurface at value of the 0.004 Å^{-3} (right). The green (orange) color denotes loss (accumulation) of electrons in graphene and QDTP upon combining them. According to ΔQ curve, 0.293e per molecule is transferred from graphene

to QDTP.

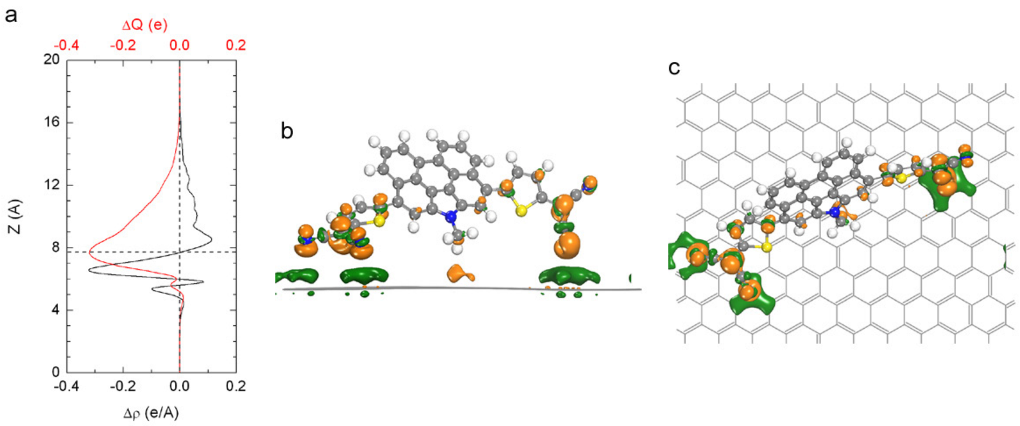

Supplementary Figure 14. **Charge transfer between graphene and QDTP molecule in triplet state in stand-up configuration.** Left panel (a): Plane-averaged differential charge density $\Delta\rho(z)$ (black line), amount of transferred charge ΔQ (red line). The horizontal dashed line in the ΔQ curve defines the maximum amount of transferred charge from the molecule to the graphene. Middle (b) and right (c) panels: Side and top views of the isosurface at value of the 0.004 $Å^{-3}$ (right). The green (orange) color denotes loss (accumulation) of electrons in graphene and QDTP upon combining them. According to ΔQ curve, 0.32e per molecule is transferred from graphene to QDTP.

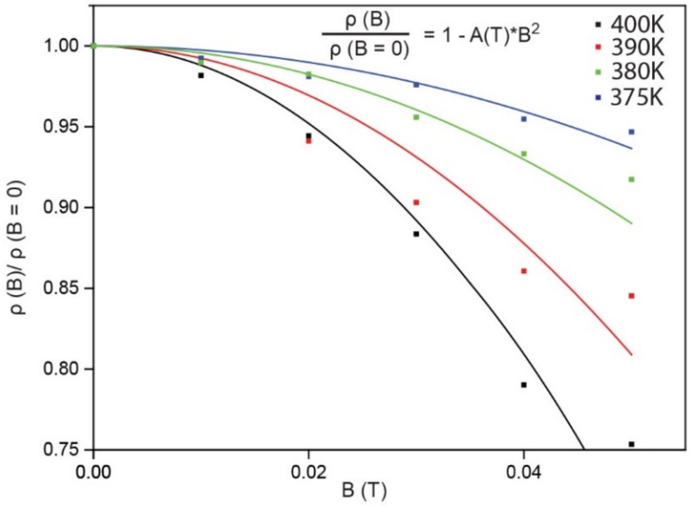

Supplementary Figure 15. **Fitting of magnetoresistivity data of MoS_2 – QDTP hybrid above spin transition temperature.** Normalized spin-order scattering resistivity $\rho(B)/\ \rho(B = 0)$ due to exchange interaction between triplet spin of the QDTP molecule and carrier of MoS_2 is given by: $1 - A(T)*B^2$. Here, $A(T)$ is used as a temperature dependent fitting parameter.

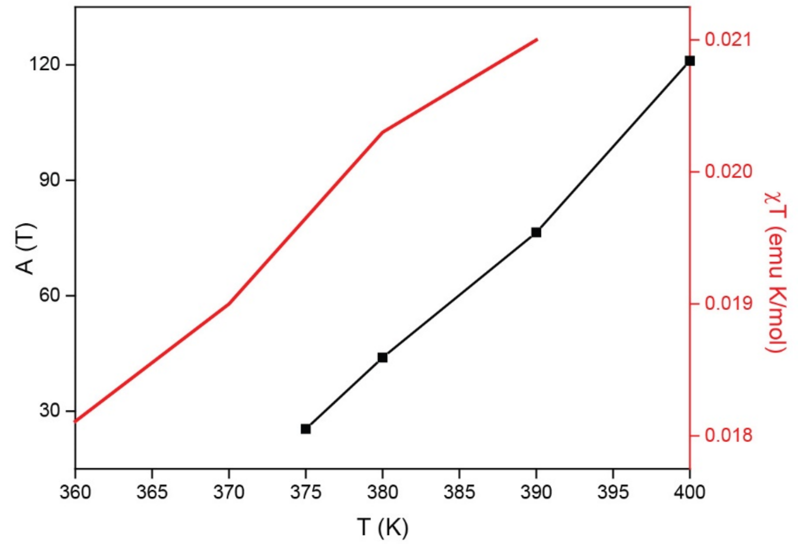

Supplementary Figure 16. **Significance of *A*(*T*).** *A*(*T*) shows similar behaviour as the temperature dependent magnetic signal (χT) of the QDTP molecule above spin transition temperature. Considering the exchange interaction between the spin triplet of the molecule and the carriers of MoS_2, one can deduce the spin-disorder resistivity as discussed in previous figure [2]. In that equation, *A*(*T*) contains the square of the thermal average of the spin, <**S**>, of QDTP molecule. <**S**> can be calculated from the SQUID data (χT *vs* T plot in red) of the molecule at high temperature [3]. The qualitative agreement of *A*(*T*) and χT, with good fitting of the resistivity data, confirms the presence of magnetic interaction mediated by carriers in MoS_2 – QDTP hybrid.

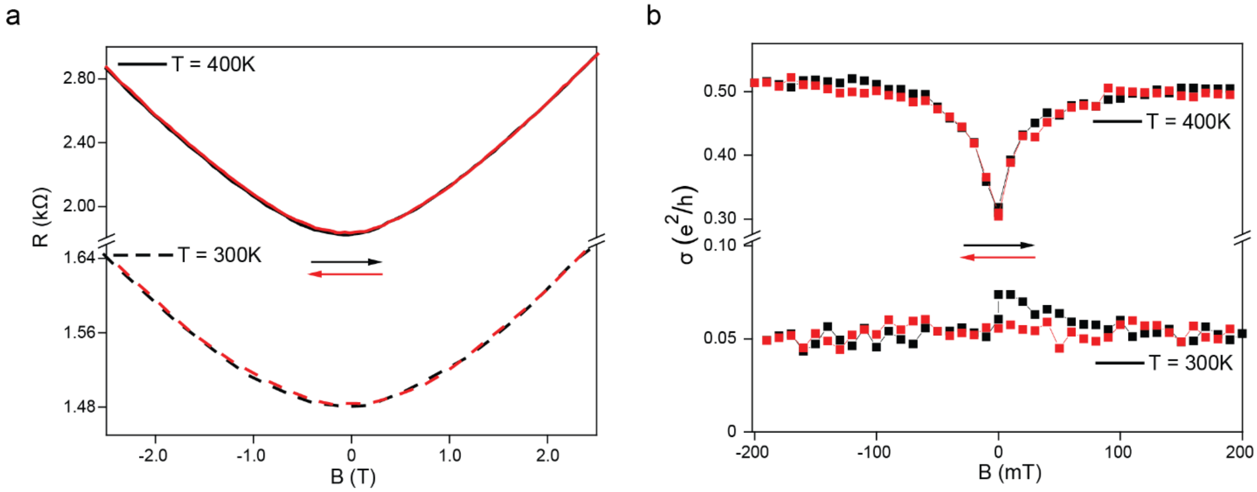

Supplementary Figure 17. **Hysteresis loop as a function of field**. Both hybrids (graphene-QDTP, MoS2-QDTP) show no hysteresis in resistance as magnetic field is swept back and forth

for two different temperatures, 300 K (singlet state of QDTP), and 400 K (triplet state of QDTP). This suggests that there is no anisotropy in the QDTP molecule which can cause hysteresis.

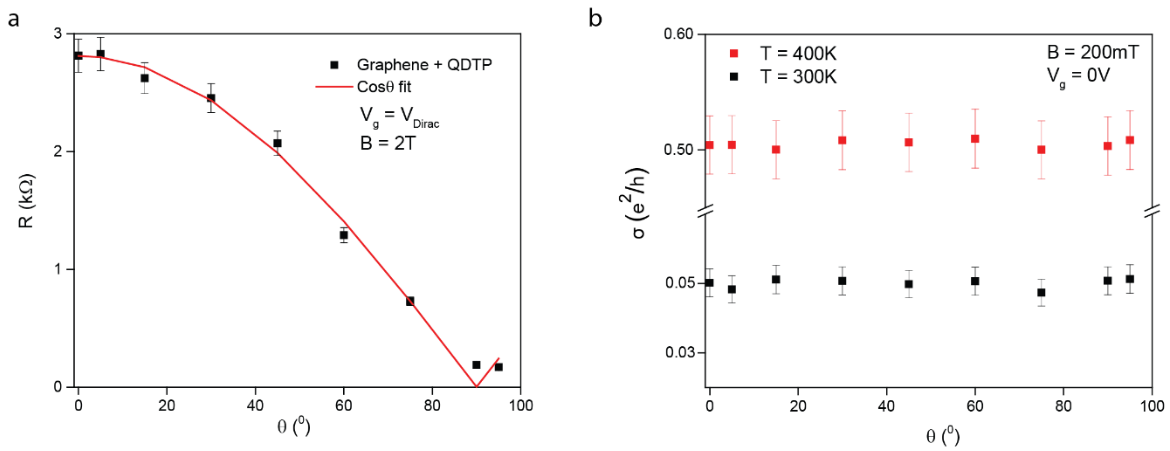

Supplementary Figure 18. **Angle dependent magnetic field effect on conductance**. Out-of-plane magnetic field is 0°, while in-plane is 90°. **a** graphene-QDTP hybrid has cosine θ dependence with magnetic field similar to graphene. **b** MoS2-QDTP has no orientation dependence with the direction of the magnetic field, for both singlet and triplet states of the molecule.

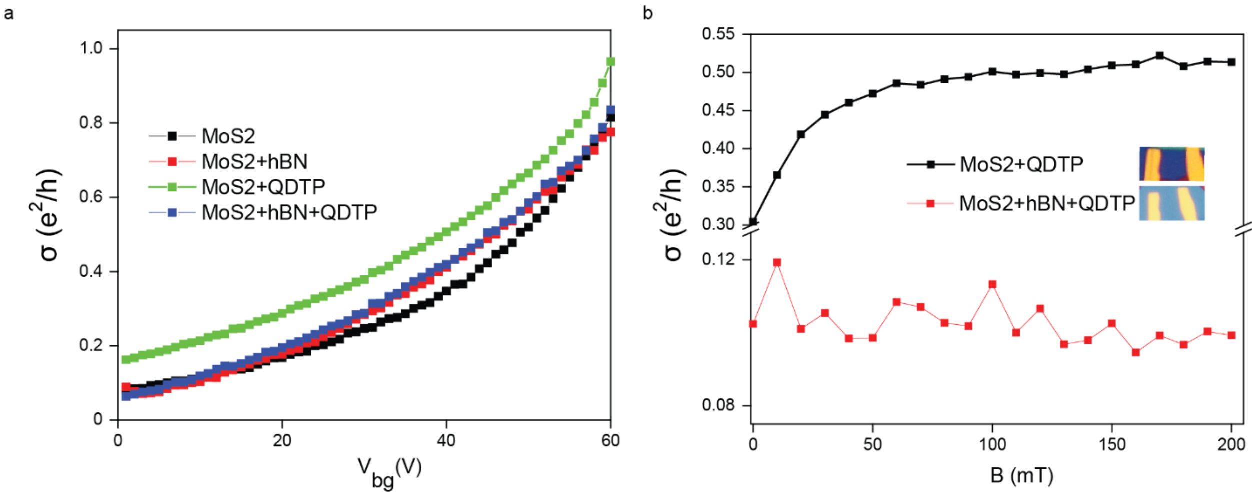

Supplementary Figure 19. **Decoupling exchange coupling between molecular spin and MoS_2.** **a** h-BN blocks the charge transfer from QDTP to MoS2. Unlike MoS_2-QDTP, no change of

transfer curve occurs after doping with QDTP in MoS2-hBN-QDTP device. b In case of MoS2-hBN-QDTP, no magnetoconductance is observed at 400K. Introducing a spacer layer like hBN in between MoS2 and QDTP completely decouples the magnetoconductance effect at high temperature, which evidences the presence of exchange coupling in the MoS2-QDTP device above the spin transition temperature of the molecule.

Supplementary Note 1

Calculation of exchange coupling constant *J*

The coupling constant *J* can be estimated from DFT calculations using the energy difference between the spin triplet and singlet states (ΔE ~ 0.36 eV). The theoretical values of *J* based on the current structural model is estimated to be around 131 eV $Å^2$. It is expected that this value is sensitive to the packing configurations of the adsorbing QDTP molecules above the 2D sheets. We have also estimated *J* to be 200 eV A^2 from the experiment, following equation (3) of Supplementary Reference 2:

$$J = [\frac{1}{2\pi^2}\rho\frac{n\,e^2}{k_F}\frac{h^3}{m^2}\frac{1}{n_s}\frac{1}{[S(S+1)- <S>^2]}]^{1/2} \qquad (1)$$

Here, we have used $S = 1$ (as triplet state), n is the electron density in MoS_2 which is estimated to be $0.6\text{x}10^{21}$e per cm^2, ns is the doping concentration of the molecule which is estimated to be around $0.27\text{x}10^{18}$ m^{-2}, m is the effective mass ~ $0.4m_e$, and <*S*> calculated from the SQUID measurement. It is noted that the n_s can vary up to orders depending on the doping concentration and the packing density of the molecules. Thermal average of <*S*> can be found from the SQUID measurement.

We have also estimated *J* (considering intermolecular spin interaction) based on experimental

measurement of the susceptibility of the molecules, and it turns out to be 100 meV, much smaller than spin-electron exchange coupling (140 eV).

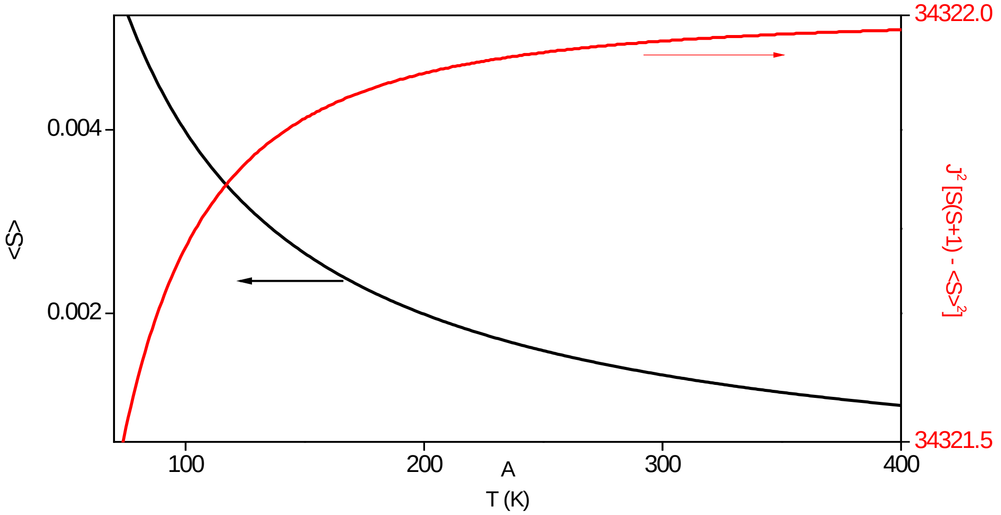

Supplementary Figure 20: **Temperature dependent spin imbalance.** Plot of thermal average of spin (<*S*>), and J^2 [*S*(*S*+1) - <*S*>^{2}] as a function of temperature (*T*), calculated using Boltzmann statistics. Qualitatively, a small fraction of <*S*> could contribute to a large MR if the exchange interaction (*J*) is large, as happened in our hybrid MoS_2 device.

Supplementary References:

[1] Tongay, S. *et al*. Defects activated photoluminescence in two-dimensional semiconductors: interplay between bound, charged, and free excitons. *Sci. Rep.* **3**, 2657 (2013).

[2] Matsukura, F., Ohno, H., Shen, A. & Sugawara, Y. Transport properties and origin of ferromagnetism in (Ga,Mn)As. *Phys. Rev. B* **57**, R2037(R) (1998).

[3] Zeng, Z. *et al*. Turning on the biradical state of tetracyano-perylene and quaterrylenequinodimethanes by incorporation of additional thiophene rings. *Chem. Sci.* **5,** 3072-3080 (2014).
